# *In Situ* Rhizosphere Microbiome in Antarctic Vascular Plants, Modulated by Soil Condition

**DOI:** 10.1128/MRA.01125-20

**Published:** 2020-12-10

**Authors:** Claudia Rabert, Giovanni Larama, Alejandra Fuentes, Ana Gutiérrez-Moraga, Daisy Tapia-Valdebenito

**Affiliations:** a Instituto de Ciencias Biomédicas, Facultad de Ciencias de la Salud, Universidad Autónoma de Chile, Temuco, Chile; b Centro de Modelación y Computación Científica, Universidad de la Frontera, Temuco, Chile; c Laboratorio de Biorremediación, Facultad de Ciencias Agropecuarias, Universidad de La Frontera, Temuco, Chile; d Instituto de Ciencias Biomédicas, Facultad de Ciencias de la Salud, Universidad Autónoma de Chile, Santiago, Chile; Georgia Institute of Technology

## Abstract

Antarctic soils are considered young soils; therefore, the microbiota associated with Antarctic vascular plants play a critical role in their productivity. In this research, we compared the microbiota from three different soil conditions using a 16S rRNA and internal transcribed spacer rRNA gene amplicon approach for bacterial and fungal communities.

## ANNOUNCEMENT

The Antarctic Peninsula territory has been naturally colonized by only two vascular plants, *Deschampsia antarctica* and *Colobanthus quitensis* (1). In this research, we study the compositions and variations of bacterial and fungal communities associated with Antarctic plants under three different cover compositions, i.e., S1 (100% vascular plants), S2 (vascular plants mixed with moss), and S3 (vascular plants and uncovered soil).

The samples were randomly collected during the Antarctic summer of 2019 from different locations near Admiralty Bay on King George Island, Antarctic Peninsula (S1, 62°09.738′S, 58°27.982′W, at 25 m above mean sea level [mamsl]; S2, 62°09.820′S, 58°28.168′W, at 59 mamsl; S3, 62°09.861′S, 58°28.357′W, at 170 mamsl). The top 20-cm depth of three rhizosphere soils, containing both plant species in coexistence, was sampled at each site. The root-attached soil was considered the rhizosphere soil and was sampled after gentle shaking of the roots. The samples were homogenized, deposited in cryotubes, and immediately preserved in liquid nitrogen. Total rhizosphere soil DNA of each subsample was isolated using a MO BIO kit for soil samples and delivered to Novogene (Sacramento, CA, USA) for paired-end sequencing on an Illumina MiSeq platform for 250 cycles. The sequencing was performed with primers 341F and 806R, aiming for the V3-V4 region of the bacterial 16S rRNA gene, and primers ITS3-2024F and ITS4-2409R, aiming for the internal transcribed spacer 2 (ITS2) region in fungal communities. All of these amplicons were obtained according to Novogene standard procedures.

The sequencing outputs were 1,502,777 and 1,511,643 paired-end reads for the 16S rRNA and ITS amplicons, respectively, with all of the samples having no less than 155,000 paired-end reads. These reads were further processed in the QIIME2 environment (2); an error correction model approach was used with the DADA2 algorithm (3) implemented in QIIME2, resulting in an average of 1,298 amplicon sequence variants (ASVs) identified in the 16S rRNA amplicons and an average of 437 ASVs identified in the ITS region amplicons. The resulting ASVs were assigned for taxonomy using the naïve Bayes classifier in the QIIME2 environment, with the Greengenes v13.8 (4) and UNITE (5) databases being used as references for the 16S rRNA and ITS amplicons, respectively. Default parameters were used for all of the software contained in the QIIME2 environment.

The taxonomic assignment results showed that *Proteobacteria*, *Actinobacteria*, and *Firmicutes* were the most abundant phyla (>10%) shared among all of the analyzed sites; *Proteobacteria* was the most abundant at site 2 (>30%), *Actinobacteria* at site 3 (>24%), and *Firmicutes* at site 1 (>17%). In addition, *Ascomycota* was the most abundant fungal phylum at the analyzed sites, with an abundance of 48.98% ± 7.44% for site 1, 37.06% ± 18.09% for site 2, and 26.40% ± 15.88% for site 3. It is relevant to mention that large percentages of fungal ASVs (site 1, 47.7%; site 2, 57.52%; site 3, 68.27%) were not able to be assigned using the UNITE database. All of these important differences may play significant roles in the capacity of *D. antarctica* and *C. quitensis* to adapt and colonize different soil conditions.

### Data availability.

The raw data have been deposited in the Sequence Read Archive (SRA) with the BioProject accession number PRJNA635264.
